# Effects of Hysterectomy on Sexual Function

**DOI:** 10.1007/s11930-014-0029-3

**Published:** 2014-09-14

**Authors:** Risa Lonnée-Hoffmann, Ingrid Pinas

**Affiliations:** 1 Institute of Public Health, St. Olav’s Hospital, Norwegian University of Science and Technology, Trondheim, Norway; 2 ZBC FeM-poli, Zwolle, The Netherlands; 3 Faculty of Applied Psychology, University of Applied Sciences, Leiden, The Netherlands

**Keywords:** Gynecological surgical procedures/adverse effects/methods, Hysterectomy, Outcome assessment (health care), Sexual behavior/physiology/psychology, Sexual dysfunction/physiological/etiology, Sexual function, Women’s health

## Abstract

Hysterectomy remains the most common major gynecological surgery. Postoperative sexual function is a concern for many women and their partners. In this respect, a beneficial effect of hysterectomy for benign disease independent of surgical techniques or removal of the cervix has been demonstrated in the past decade by the majority of studies. For about 20 % of women, deteriorated sexual function has been reported and current research is attempting to identify mechanisms and predictive factors explaining these postoperative changes. Alternative treatments of benign uterine disorders or uterus preserving surgery for genital prolapse appeared to have similar outcomes in terms of sexual function. Concomitant oophorectomy had negative effects on sexual function and long-term health, particularly in premenopausal women. This may not be reversed by estrogen replacement. Hysterectomy performed for malignancy had a detrimental effect on sexual function. Individualized risk assessment and information should be aimed at during preoperative decision making.

## Introduction

Hysterectomy is defined as the removal of the uterine corpus with (total hysterectomy) or without the cervix (subtotal or supra cervical hysterectomy). The route can be via laparotomy, vaginally, by applying minimally invasive techniques (laparoscopy, robotic surgery) or a combination of the latter two. Hysterectomy is the most common major surgical procedure performed among women in the USA after cesarean section [1]. The indications include conditions like bleeding problems, uterine leiomyoma, endometriosis and uterine prolapse, and malignant conditions of the internal genital tract. Concomitant bilateral oophorectomy is performed in about 50 % of all cases [1, 2]. This reduces the risk of ovarian cancer to almost zero; however, the resultant estrogen deficit may have more detrimental effect on women’s health [2].

In the context of this review, the term “sexual function” is used as an overall descriptive term for outcomes based on validated and non-validated instruments. This includes sexual activity and sexual function in terms of specific functional aspects as well as satisfaction with sexual activity.

Hysterectomy performed to alleviate symptoms based on somatic conditions in general improves female sexual function and quality of life, according to reviews within the past decade [3–5]. The improvement seems to be independent of the surgical route or whether the cervix is removed or not (level 1B evidence) [6, 7]. In African and Asian populations, a similarly positive outcome has been confirmed [8, 9].

Yet, reports of sexual function following hysterectomy are inhomogeneous. One explanation for this is incomparable study populations with different psychological and endocrine conditions, more specifically, pre or postmenopausal women with or without depression, and with or without bilateral oophorectomy (BSO). Differing study designs add to comparison difficulty. For instance selection bias may be present in observational studies. Kupperman et al. reported systematic differences between women opting for hysterectomy compared to conservative treatment [10]. As a positive developement in terms of study design, in the past decade, a number of randomized controlled trials (RCTs) on the topic of hysterectomy including sexual function have been published including a Cochrane review [11••, 12–14, 15••]. Another complicating factor is the multidimensional nature of female sexual function, in particular as they age, women’s sexual function and well-being is affected in addition to biological and psychological factors by relationship, social, and cultural aspects [16].

A decidedly positive development is that the proportion of studies using validated instruments to measure sexual function has been further increasing.

Concerns about sexual function are an important cause of anxiety for women undergoing hysterectomy [17]. Indeed, 21 % of women with a mean age of 59 stated that the uterus is important for their sexual experience and after hysterectomy 10–20 % may experience deterioration of their sexual function [18, 19]. Important current topics of research are how sexual function is affected by hysterectomy and to identify predictors for improvement or deterioration of sexual function. Preoperative sexual problems and/or depression have been shown to be predictive factors for reduced desire [19–21]. Likewise, women (online convenience sample) reporting endometriosis to be the reason for hysterectomy demonstrated less improvement of sexual function, compared to women with other (benign) indications [22]. The authors of a recent review article summarized possible pathways for deleterious physical effects of hysterectomy: scar tissue in the vagina might prevent full ballooning of the upper vagina, removed tissue may reduce the capacity for vasocongestion and with or without nerve damage, this could reduce arousal or cause dyspareunia [21]. Vaginal length was not related to sexual function [23]. Experimental evidence confirmed that hysterectomy caused sensory loss in the vagina, without impacting sexual function [24]. Studies investigating an association between postoperative decreased elasticity and sexual function are lacking.

It has been hypothesized that women for whom uterine/cervical contractions are an important aspect of orgasm, may be a subgroup more likely to experience reduced sexual function after hysterectomy [21]. The debate is ongoing, if and to which degree there is more than one type of orgasm. In this context, it may be important to differentiate between the site of stimulation and the site of perception of orgasmic sensations [25]. Currently, there is a lack of studies, applying validated and sensitive instruments to investigating the impact of (the type of) hysterectomy on orgasmic function.

Interestingly, it has been demonstrated that patient education about potential negative sexual outcomes after hysterectomy (but not positive outcomes!) may enhance satisfaction with the procedure, independent of whether sexual problems were experienced or not [26].

Further research in the past decade about effects of hysterectomy on sexual function can be grouped under the subheadings following below.

## Total Versus Subtotal Hysterectomy

In 2012, a comprehensive Cochrane review was published, comparing subtotal with total hysterectomy for benign indications [11••]. Six randomized controlled trials (RCT) (performed between 2002 and 2010) had outcomes related to sexual function, with a follow-up time for up to 2 years; one of these studies compared laparoscopic routes. The authors found no evidence for difference in sexual satisfaction, or patient-reported dyspareunia between subtotal and total hysterectomy in their meta-analysis. The authors of the Cochrane review commented on a lack of blinded studies, causing a degree of uncertainty with regard to subjective outcomes such as sexual function.

One of the RCTs included in the Cochrane review, recently completed a 5-year follow-up, and still without finding a difference in sexual satisfaction for the two types of hysterectomy [12].

In a recently published, observational study, women after subtotal laparoscopic hysterectomy (*n* = 788) reported more often improved sexual function as well as sooner resumption compared with total laparoscopic hysterectomy (*n* = 127) [27]. Female sexual function index (FSFI) scores did not differ between the two groups.

In summary, currently, there is no good evidence to support the notion that subtotal hysterectomy may result in better postoperative sexual function, compared with total hysterectomy. Intercourse is likely to be resumed earlier after subtotal hysterectomy.

## Vaginal Hysterectomy or Hysterectomy for Pelvic Organ Prolapse

Increased sexual activity was reported among 20 % of women 6 months after vaginal hysterectomy (VH) (*n* = 791) in a retrospective Swedish study [28].

When comparing VH plus uterosacral ligament suspension (*n* = 38) with robotic hysterectomy plus colpopexy (*n* = 46), the authors found no difference in sexual function, despite postoperative shorter vaginas in the VH group [29]. Neither did they find any significant change of postoperative sexual function, assessed by a condition-specific sexual function questionnaire.

A number of studies compared different types of uterus-sparing surgery with prolapse surgery plus hysterectomy. One follow-up study from Italy compared uterus-sparing surgery (hysterosacrocolpopexy) (*n* = 32) with sacrocolpopexy plus hysterectomy (*n* = 36) [30]. Sexual function, measured with the FSFI improved in both groups, but greater improvement was observed among women with preserved uterus. These results should be interpreted with caution because BSO had been performed in the hysterectomy group in addition, which could be an important confounding factor. These results are in contrast to prior findings from the same group, when no significant differences in FSFI scores were reported after uterus-sparing surgery (*n* = 15) compared with sacrocolpopexy plus hysterectomy (*n* = 22); in the latter study, no BSO had been performed, which complicates comparison [31]. Women 4–9 years after the Manchester Fothergill procedure (removal of cervix and anterior colporrhaphy (*n* = 98) did not differ with regard to sexual function, compared with women after vaginal hysterectomy (VH) (*n* = 98) in another observational study [32].

In summary, sexual function after VH is likely to be unchanged or improved for most women. Alternative procedures for uterus prolapse, involving uterus-sparing surgery, seem to have comparable outcomes with respect to postoperative sexual function.

## Hysterectomy and Bilateral Oophorectomy

Studies on hysterectomy and elective bilateral oophorectomy previously have mainly focused on cancer risk reduction and general health issues rather than sexual function. In women requiring hysterectomy for benign disease, preventive removal of the ovaries may be routinely offered to reduce risk of cancer or other adnexal pathology. In the USA, oophorectomy occurs in almost half of benign hysterectomies because of perceived risk of breast or ovarian cancer, anxiety, or financial reasons [33••]. Since lifetime risk of 1.4 % for ovarian cancer seems relatively low, benefits of preventive oophorectomy may not outweigh risks. Increased perioperative complications, e.g., organ injury, circulatory, bleeding, or gastrointestinal complications have been reported with BSO and hysterectomy [33••]. Women may be assured that hysterectomy is usually associated with improved quality of life and sexual function, but concomitant oophorectomy has been shown to compromise long-term outcomes depending on the age at which the procedure was performed. After hysterectomy with BSO, a 20 % breast cancer risk reduction has been demonstrated in a large prospective study (*n* = 66,802) from the USA [34]. Yet, no reduced risk of all-cause mortality at any age has been demonstrated in another large, prospective US study (*n* = 30,117), due to higher mortality from cardiovascular disease and lung and colon cancer [35••]. Ovarian removal at premenopause had significant negative impact on cardiovascular, cognitive, mental, and psychosexual health [36]. The resultant estrogen and androgen deficiency leads to more aggravated climacteric symptoms and sexual dysfunctions, e.g., decrease in sexual pleasure, comfort, and frequency. In postmenopausal women, overall sexual function (sexual desire, vaginal lubrication, intimacy-seeking behavior) and FSFI scores decreased after oophorectomy [33••]. Sexual infrequency and multiple sexual function problems have also been reported with increased level of menopause symptom intensity when comparing surgical and natural menopause [37]. Indeed, lower desire, arousal, lubrication, and sexual satisfaction, besides more coital pain, are frequent sexual complaints after perimenopausal oophorectomy.

Estrogen replacement therapy may enhance lubrication and reduce vasomotor symptoms, it may not suffice to improve overall sexual function [38].

To summarize, combining hysterectomy with bilateral oophorectomy may result in impaired sexual function and increased perioperative and long-term health risks, with benefits only for women at high risk of ovarian cancer. Estrogen replacement may not sufficiently improve sexual function and general health. Individualized risk assessment and information should aid in preoperative decision making.

## Alternative Treatments Compared to Hysterectomy

The Society for Gynecologic Surgeons published a systematic review in 2012, to compare hysterectomy to alternative treatments for abnormal uterine bleeding (AUB) [39]. Included were nine RCTs, comparing endometrial ablation (EA), the levonorogestrel intrauterine system (Lng-IUS), and systematically administered medical therapy (different types of estrogen and/or progesterone preparations). Based on this review, clinical practice guidelines were issued, with recommendations graded either strong or weak [40•]. Concerning sexual function, recommendations were weak and no advantage of any of these methods over hysterectomy was found. Conclusion was, that the choice of treatment should be based on additional patient preferences. In the review, one RCT comparing Lng-IUS to hysterectomy was included, showing no significant reduction in sexual problems after 5 years in the two groups. However, the same RCT also reported maintained improvement of sexual satisfaction after 5 years among hysterectomized women, while it was reduced among Lng-IUS users after both 1 and 5 years [13]. Findings of a large observational study following 8,900 mostly premenopausal women for up to 5 years after hysterectomy or EA are in contrast to results of the systematic review [41]. The authors found a higher crude and adjusted prevalence of psychosexual problems among hysterectomized women; however, non-validated questionnaires were used.

Uterine artery embolization (UAE) (*n* = 88) was compared to hysterectomy (without BSO) (*n* = 89) in a Dutch RCT [14]. Significantly improved sexual function was only observed after UAE; a validated questionnaire was used.

Gonadotropin-releasing hormone agonist (GnRHa)—treatment (*n* = 42) as an alternative to hysterectomy (*n* = 50)—was examined in an observational study in premenopausal women above age 45 [42]. After 2 years, no significant differences in FSFI scores (domain or total) were observed.

Finally, an interesting study from the USA included 1,492 premenopausal women presenting with AUB, chronic pelvic pain, or symptomatic leiomyoma and followed them for a mean period of 3.6 years [43]. The objective was to observe long-term effects of three defined interventions—hysterectomy (14 %), uterus-preserving surgery (9 %), and no surgery (77 %). The scores for the pelvic-problem-impact-on-sex scores improved in the long term for all three outcome groups; most improvement was observed after hysterectomy.

In summary, most evidence collected over the past decade show similar improvements in sexual function for alternative treatments to hysterectomy for benign disease. Different options with defined risks and benefits should be discussed with the woman and her preferences considered.

## Hysterectomy for Cervical Cancer

As a consequence of cervical cytology screening, most women diagnosed with cervical cancer are stage 1, with an expected 5-year survival of at least 90 % in a Nordic setting [44]. Sexual problems and dysfunction are common after radical surgery for cervical cancer; particularly, lower desire and inadequate lubrication are reported to persist 2 years after surgery, while other sexual problems decrease after this period [45]. Explanations are suggested to be damage to the local autonomous nervous system, resection of the paracolpium, shortening of the vagina, and loss of ovarian function [46] Individualization adapted to patient and tumor-related factors is the current trend in cervical cancer surgery to minimize therapy-associated morbidity [46]. As the first choice, it is recommended to preserve nerves with minimum damage, yet without reducing radicality (type C), as the second choice to preserve nerves using less radical resection techniques (type B) [46]. Nerve-sparing radical hysterectomy has been shown to have less negative effect on sexual function than RH [47].

Radical vaginal trachelectomy (RVT) has been an option for nearly two decades for patients with stage A1 IB1, tumor size less than 2 cm, and a desire to preserve fertility. An observational study from Denmark compared sexual function among three groups: 1 year after RVT (*n* = 18, median age 29 years) and after radical hysterectomy (RH) (*n* = 32, median age 42 years) to a healthy control group (*n* = 30, median age 29 years) [48]. Postoperative sexual activity increased in the RVT group during the follow-up and reached that of the control group after 1 year. However, in the RVT group, sexual distress measured with the Female Sexual Distress Scale (FSDS) was significantly higher and FSFI scores significantly worse, after adjusting for confounders, compared with women both after RH and in the control group. Similar, somewhat disappointing results with regard to RVT were confirmed by two other studies [49, 50]. One of these studies compared RH, RVT, and cervical conisation for early-stage cancer and found only on the conisation group no increase in the proportion of women with sexual dysfunction [50].

Likewise, vaginal extension (*n* = 31) failed to result in improved sexual function, compared to RH (*n* = 28) without extension in a case-control study, despite an average difference of about 4 cm [51]. Finally, laparoscopic radical hysterectomy (*n* = 20) fell short of demonstrating a beneficial effect on postoperative sexual function as well, compared with abdominal hysterectomy (*n* = 18), in a case-control study [52].

Referral for supportive oncosexology care, a new therapeutic offer aiming to improve sexual health of cancer patients may be indicated, although no studies have been published as yet, demonstrating their effectiveness [53, 54].

In summary the high proportion of long-time survivors after cervical cancer may benefit from the increased amount of research focusing on less-invasive surgical procedures with potentially less detrimental effect on sexual function for selected patients. Research in this field is hampered by the lack of RCTs. No clear benefit in terms of improved sexual function has been demonstrated for RVT, but seems likely for nerve-sparing techniques and for individualized oncosexology care.

## Hormone Treatment After Hysterectomy

Among postmenopausal, hysterectomized women, estrogen therapy (ET) by vaginal route (*n* = 30) was compared with systemic administration (*n* = 27) in a RCT from Taiwan, assessing sexual function with a validated questionnaire [55]. Vaginal blood flow improved and anorgasmia decreased with both forms of ET application, but vaginal dryness and dyspareunia significantly improved only with vaginal ET. Desire or frequency of activity did not improve. An observational study from Turkey compared four groups of postmenopausal women after hysterectomy plus BSO: VH, using ET (*n* = 21), VH no ET (*n* = 16), abdominal hysterectomy using ET (*n* = 28), and abdominal hysterectomy no ET (*n* = 27) [56]. FSFI scores deteriorated in all groups, except after VH and ET use. Type of ET use was not defined [56].

Testosterone therapy for postmenopausal, hysterectomized women (*n* = 71) was associated with improved desire and arousal and increased frequency of sexual activity in a RCT from the USA [15••]. This improvement was significant only for the group with the highest testosterone dose (25 mg testosterone enanthate weekly, intra muscular). Sexual function had been assessed with a validated questionnaire and some women had undergone concomitant BSO.

In summary, ET, in particular vaginal application, is likely to benefit some aspects of sexual function after hysterectomy with BSO. This may also be the case after testosterone treatment, although this should be confirmed by more studies.

## Vault Dehiscence After Hysterectomy

This complication has been more commonly reported in the last decade, in particular after robot-assisted total hysterectomy. Based on 441 women following robotic hysterectomy for gynecological cancer, a risk of 3 % was calculated for vault dehiscence in combination with chemo or brachytherapy and 0.4 %, if no adjuvant therapy was given [57]. All women had been operated at one institution. Another associated risk factor for vault dehiscence was early resumption of intercourse. After laparoscopic hysterectomy for benign indication, a risk of 0.6 % was estimated, including 677 women at another institution [58]. Sexual intercourse after 47–103 days post surgery had been the triggering event in most cases. A large multicenter study reviewed the charts of nearly 9,000 women, one third each after abdominal, vaginal, and laparoscopic hysterectomy [59]. The overall risk for vault dehiscence was 0.4 % and highest after laparoscopic surgery with intercourse before completed healing of the vaginal cuff identified as the main trigger among young patients.

Recommendations have been made to extend the period for abstinence from deep penetrative intercourse after minimally invasive total hysterectomy to 8–12 weeks, although dehiscence after penetration had occurred as late as 4 months after surgery [60].

In summary, vault dehiscence is rare after surgery for benign disease and an incidence of about 3 % is reported, if laparoscopic surgery for malignancy was followed by adjuvant therapy. Early resumption of intercourse was identified as a risk factor. Women need to be informed about this complication and that abstinence from deep penetration may be advisable for the first 3 postoperative months. Further evidence confirming this recommendation is needed.

## Hysterectomy and Partner Sexual Function

During the past decade, there continues to be a lack of research with a focus on partner experience in the context of hysterectomies.

This despite the findings of two qualitative studies, one from China, the other from Brazil, that men had considerable concerns about changes in postoperative sexuality or sexual abstinence around the time of hysterectomy of their partner [61, 62].

Unchanged or improved sexual satisfaction for men, with no significant difference after total abdominal hysterectomy (*n* = 60) or subtotal abdominal hysterectomy (*n* = 60), has been reported in a retrospective study from Norway [63]. After subtotal hysterectomy, more men noticed during intercourse that the uterus had been removed, but none of these partners experienced this as negative.

In summary, for the male partner, sexual function after benign hysterectomy appears to be an important issue and some evidence exists that men can also expect unchanged or improved sexual satisfaction, regardless of removal of the cervix.

## Conclusion

In this review, we summarized literature from the past 10 years regarding effects of hysterectomy on sexual function. Current evidence suggests that hysterectomy for benign disease has beneficial effects on sexual function and general well-being irrespective of the surgical technique used. About 10–20 % of women may experience deteriorated sexual function, for example, dyspareunia or altered orgasmic experience. Risk factors for postoperative sexual dysfunction are preexistent psychiatric morbidity like depression and unsatisfactory sexual function. Health care providers should inform women accordingly.

Hysterectomy indicated by gynecologic malignancy has complex consequences and associated worsening of sexual function. Lower desire and inadequate lubrication are most persistent. For very-early-stage disease, new techniques to preserve sexual function are promising but more RCTs needed. Care providers focusing primarily on management of malignant disease should consider referral for specialized oncosexology counselling.

Preventive bilateral oophorectomy at the time of hysterectomy for benign disease has detrimental effects on various effects of sexual function and long-term health and has been associated with increased mortality. Estrogen replacement therapy after hysterectomy may reduce complaints of lubrication and dyspareunia; however, it may not suffice after concomitant premenopausal oophorectomy. Additionally, the sharply declining hormone use of the past decade raises critical concerns in this context [64]. Individualized risk assessment and information should aid in preoperative decision making, also with respect to BSO.

Vault dehiscence has been reported as a complication after 3 %, in particular robot-assisted total hysterectomies for malignancy, followed by adjuvant therapy. The most common precipitating factor appears to be intercourse, and as a practice-based preventive measure, it is recommended to abstain from intercourse for about 3 months after hysterectomy for malignant disease.

Sexual function of the partner after hysterectomy has been insufficiently addressed in the scientific literature, and we did not find reports about sexual function after emergency peripartum hysterectomy.

In conclusion, hysterectomy ultimately eliminates bleeding problems, coital pain, and contraception-related issues, which may all contribute to better quality of life and sexual function. Evidence-based strategies to prevent or minimize postoperative deterioration of sexual function will benefit women facing hysterectomy.
